# Identification of modified peptides using localization-aware open search

**DOI:** 10.1038/s41467-020-17921-y

**Published:** 2020-08-13

**Authors:** Fengchao Yu, Guo Ci Teo, Andy T. Kong, Sarah E. Haynes, Dmitry M. Avtonomov, Daniel J. Geiszler, Alexey I. Nesvizhskii

**Affiliations:** 1 Department of Pathology, University of Michigan, Ann Arbor, Michigan USA; 2 Department of Computational Medicine and Bioinformatics, University of Michigan, Ann Arbor, Michigan USA

**Keywords:** Peptides, Mass spectrometry, Proteomics, Proteome informatics

## Abstract

Identification of post-translationally or chemically modified peptides in mass spectrometry-based proteomics experiments is a crucial yet challenging task. We have recently introduced a fragment ion indexing method and the MSFragger search engine to empower an open search strategy for comprehensive analysis of modified peptides. However, this strategy does not consider fragment ions shifted by unknown modifications, preventing modification localization and limiting the sensitivity of the search. Here we present a localization-aware open search method, in which both modification-containing (shifted) and regular fragment ions are indexed and used in scoring. We also implement a fast mass calibration and optimization method, allowing optimization of the mass tolerances and other key search parameters. We demonstrate that MSFragger with mass calibration and localization-aware open search identifies modified peptides with significantly higher sensitivity and accuracy. Comparing MSFragger to other modification-focused tools (pFind3, MetaMorpheus, and TagGraph) shows that MSFragger remains an excellent option for fast, comprehensive, and sensitive searches for modified peptides in shotgun proteomics data.

## Abstract (web summary)

Mass spectrometry-based proteomics is the method of choice for the global mapping of post-translational modifications, but matching and scoring peaks with unknown masses remains challenging. Here, the authors present a refined open search strategy to score all peaks with higher sensitivity and accuracy.

## Introduction

Analysis of protein posttranslational modifications (PTMs) is key to understanding biological systems. Shotgun proteomics, in which proteins are enzymatically digested and analyzed via liquid chromatography–tandem mass spectrometry (LC-MS/MS), is one of the most widely used approaches to analyze PTMs^1^. Biologically relevant PTMs amenable to MS-based analysis include phosphorylation, acetylation, glycosylation, many less common modifications, and even novel PTMs that are still being discovered^2,3^. Furthermore, MS-based proteomic datasets contain a large number of peptides harboring chemical modifications introduced at the sample preparation stage^4,5^. Searching for these many possible modifications using conventional database search tools has proven impractical. As a result, a number of specialized computational strategies for identification of modified peptides have been proposed, such as alignment-based approaches, sequence tag-based methods, and spectral library searching (reviewed in 2016 by Fu^6^), including the latest generation of modification-focused tools^7–13^. Due to the large search space, most tools limit the allowed delta masses to a predefined list (e.g., UniMod^14^) and/or use a two-step search approach.

In our recent work, we introduced a fragment ion indexing strategy and an ultrafast database search engine MSFragger^15^. This advance enabled a practical implementation of the open search strategy, where large mass differences are allowed between unmodified peptide sequences and experimentally observed precursors. Owing to its fast speed and ease of incorporation into existing workflows, MSFragger and its open search strategy for comprehensive PTM analysis have quickly gained wide adoption in the proteomics community. Applications have included detection of RNA crosslinks on RNA binding proteins^16^, comprehensive PTM searching in metaproteomics studies^17^ and clinical samples^18^, quality control and checks for unexpected modifications^19^, and proteogenomics^20^.

Despite its success, the straightforward open search strategy has one significant limitation in that it does not attempt to use modification-containing (mass shifted) fragment ions in scoring. Thus, as much as half of the useful spectral information may be ignored. This is particularly true for non-labile modifications, where fragment ions retain the modification after dissociation of the precursor peptide, and especially when the modification is located in the C-terminal region of the peptide (eliminating the strongest fragment ions, *y* ions, from scoring). In addition, without using these shifted ions, the delta mass in open search could not be localized within the identified peptide. A number of studies^7,10,13,21^ tried to address this issue by using complementary peaks: transforming each experimental peak by subtracting it from the precursor mass. These complementary peaks are used together with the original peaks in matching and scoring. This approach—available as an option in MSFragger since its original release—is able to match more fragment ions^15^. However, it also increases the likelihood of false matching due to doubling the noise peaks and also generating unmatchable peaks by transforming modification-free peaks into modification-containing peaks. Tang et al.^13^ attempted to alleviate the noise problem by dividing each spectrum into small regions, and using complementary peak matching only in the spectral region deemed most likely to contain shifted ions.

Here we propose a refined open search strategy, and a shifted ion index to be used alongside the existing regular fragment ion index in scoring. In this dual indexing strategy, both shifted and regular fragments can be effectively—without increasing the noise—matched and scored to localize unknown modifications, significantly improving sensitivity and accuracy of the results. We refer to our strategy as localization-aware open search, and implement it in MSFragger as a default open search option.

## Results

### Shifted ion indexing enables localization-aware open search

**Fig. 1:**
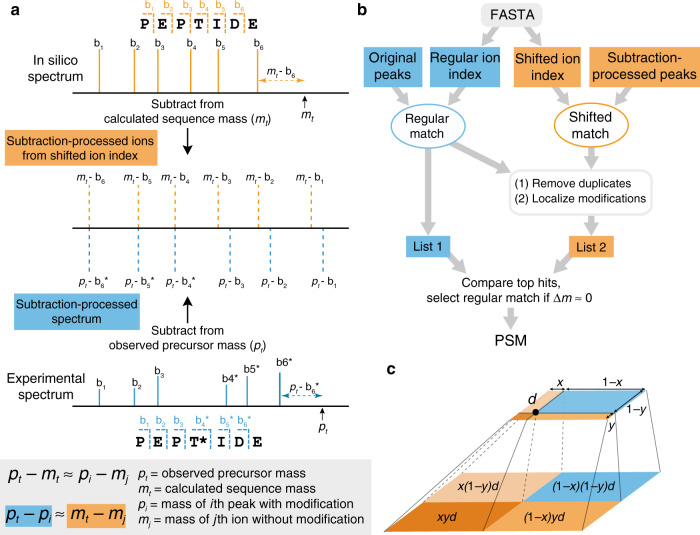
Overview of the localization-aware open search strategy. **a** Generation and use of shifted ion index using *b-*ions as an example. Each fragment peak in a theoretical spectrum (top, in orange) is subtracted from the calculated peptide mass to generate the shifted ion index. The fragment peaks can be N-terminal (e.g., *a-*, *b-*, or *c-*ions) or C-terminal (e.g., *x-*, *y-*, or *z-*ions). Each fragment peak in an experimental spectrum (bottom, in blue) is similarly subtracted from the observed precursor mass, yielding the subtraction-processed spectrum. The subtraction-processed spectrum is then compared with the shifted ion index, and fragments containing a modification (star) are matched (middle, dashed lines). **b** Overview of MSFragger search. Both shifted and regular ion indexes are generated from a sequence database. For each MS/MS spectrum, the original peaks are matched against the regular ion index to generate list 1. In the meantime, MSFragger matches the subtraction-processed peaks against the shifted ion index. If the top-scored candidate has a calculated mass significantly different from the spectrum’s precursor mass, matched peaks from the regular and shifted ion matching are combined, duplicate matches are removed, and modifications are localized using both the shifted and regular peaks to generate list 2. The top-scored PSM from list 2 is then compared with list 1, and the top-scoring hit is selected as the final hit. **c** Assigning mass errors to neighboring cells to build calibration profiles. The upper plane is a duplicate of the blue cell in the lower plane, and other three orange cells are its neighbors. The black dot in the top plane is the location of a specific peak with mass error *d*, which is assigned to the four cells weighted by the normalized distances to the boundaries (*x* and *y*).

In conventional open searching, fragment ions containing unknown modifications are not used in peak matching and scoring, and thus only half of all theoretical fragment ions can be matched to an experimental spectrum. We introduce the concept of shifted ions to describe fragment ions with unknown modifications, and call fragment ions without unknown modifications regular ions. Corresponding peaks in the experimental spectrum are called shifted and regular peaks, respectively. Shifted ions cannot be indexed by the existing method, which prevents localization and scoring of unknown modifications in open search. Here, we propose a shifted ion indexing method (Fig. 1a) that facilitates fast matching of those ions against shifted peaks, achieving localization-aware open search.

Given a theoretical spectrum, each fragment ion, including N-terminal ions (e.g., *a-*, *b-*, or *c-* ions) and C-terminal ions (e.g., *x-*, *y-*, or *z-* ions), is subtracted from the calculated peptide mass. The subtraction-processed ions are efficiently indexed to generate a shifted ion index. Peaks in each experimental spectrum are similarly subtracted from the observed precursor mass. We show in Fig. 1a and in “Methods” how matching subtraction-processed peaks against subtraction-processed ions is equivalent to matching shifted peaks against shifted ions. Note that this approach is different from the complementary ion approach that matches all peaks’ original and complementary versions against unshifted ions.

We also propose a workflow (Fig. 1b) combining shifted and regular ion indexes to facilitate fast matching to both kinds of peaks while suppressing false matches. Both shifted and regular ion indexes are generated in advance given a sequence database. Given a tandem mass (MS/MS) spectrum, MSFragger matches peaks against the regular ion index and shifted ion index separately. Then, it evaluates the top-scoring candidate from the regular ion matching only. If there is a large difference between the precursor mass and the calculated mass (by default, outside of −1.5 to 3.5 Da interval), MSFragger considers the possibility that the peptide is modified. It then combines shifted ion matches with the regular ion matches to calculate a hyperscore^15,22^ (“Methods”). The new top-scoring candidate is then compared with the old top-scoring candidate, and the highest scoring of these is picked as the final hit. For simplicity, we refer to MSFragger without shifted ion index as MSFragger regular OS, and refer to MSFragger with shifted ion index as MSFragger LOS.

Since the quality of peak matching is highly dependent on the precision of precursor and fragment masses, we developed mass calibration and parameter optimization to improve peak matching. In the following sections, we first show that mass calibration and parameter optimization help reduce random matches in both shifted and regular ion matching. Then, we demonstrate the power of localization-aware open search in terms of sensitivity, precision, and speed.

### Mass calibration and parameter optimization

In shifted ion indexing and matching, the accuracy of *p*_*t*_ − *p*_*i*_ (see Fig. 1a) is dependent on the precursor mass precision and the accuracy of peak matching is dependent on the fragment mass precision. However, some systematic mass deviation within MS and MS/MS spectra is often unavoidable and can diminish database search results. Thus, we developed a method (see “Methods”) to increase both precursor and fragment mass precision by correcting systematic mass deviation, which further increased the accuracy of matching shifted peaks. MSFragger first searches a given spectral file with a relatively small search space. Then, it picks the peptide-spectrum matches (PSMs) with expectation values smaller than 0.001 and divides them into two sets. The first set is used to build two calibration profiles (precursor mass and fragment mass) and the second set is used to evaluate the performance of the calibration. A calibration profile is a 2-D matrix of mass errors in the retention time and *m/z* dimensions. For each PSM in the first set, MSFragger calculates the precursor mass error and assigns it to the neighbors in the precursor mass calibration profile (Fig. 1c). After processing all PSMs in the first set, MSFragger corrects all PSMs’ precursor masses with the final calibration profile. It also calibrates fragment masses with a similar method (see “Methods”).

To compare the performance of our mass calibration method, we used well-known tools as benchmarks. We used eight fractions from Doll et al.^23^ (PXD006675), a dataset that displays systematic mass deviation, to compare calibration performance among MaxQuant^24^ (version 1.6.10.43), mzRefiner^25^ (from ProteoWizard version 3.0.19311 64-bit), MetaMorpheus^11^ (version 0.0.303), and MSFragger (version 2.2). MaxQuant reports mass errors (in msms.txt) for identified spectra only, while the others report mass error for all scans. Only the subset of scans identified by MaxQuant was therefore used to compare calibration performance across the different tools.

**Fig. 2:**
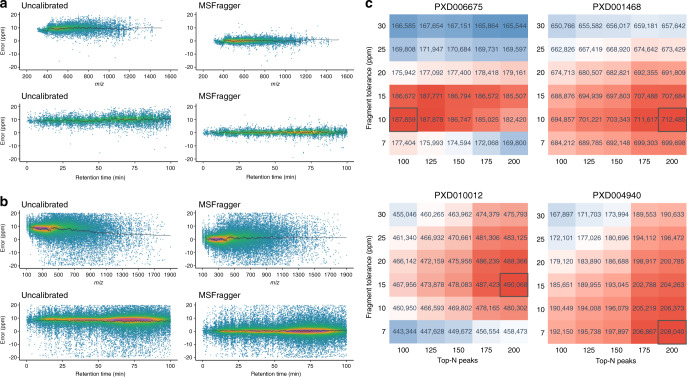
Mass calibration and parameter optimization. **a** Precursor mass error before and after mass calibration, where color indicates point density. Precursor mass error is shown against *m/z* (upper panel) and retention time (lower panel). The smoothed moving average line is shown in dark gray. **b** Same as **a**, for fragment mass calibration. **c** Parameter optimization performance of MSFragger. Each heatmap shows the number of identified PSMs as a function of the fragment mass tolerance (rows) and the number of peaks used for matching (columns). The color of each cell indicates total PSMs (at 1% FDR) compared with the benchmark search result, where parameter combinations performing better than the benchmark are colored red and those with fewer PSMs are colored blue. The optimal parameters selected by the automatic procedure are marked with a black box.

For each PSM, we calculated the relative precursor mass deviation by comparing observed mass to theoretical mass. Figure 2a displays the precursor mass deviation from one of the eight fractions across *m/z* and retention time. Other seven fractions have similar results. In each plot, the smoothed moving average line (dark gray) shows the trend of precursor mass deviation across *m/z* and retention time. Plots in the left column display the original data without mass calibration, where the observed mass deviates significantly from theoretical values. In the right column, data are shown after calibration by MSFragger. Supplementary Fig. 1a shows original mass errors and calibrated ones from all tools. All four tools (MaxQuant, mzRefiner, MetaMorpheus, and MSFragger) reduce precursor mass errors, but MSFragger and MaxQuant yielded the smallest mass errors and most uniform distributions across *m/z* and retention time.

We also calculated relative fragment mass deviation by comparing observed and calculated mass from each matched fragment peak (Fig. 2b and Supplementary Fig. 1b). Original data (left column) show large deviation in the observed fragment mass, and a nonlinear trend with respect to *m/z*. After calibration by MSFragger (right column), mass errors are corrected and much of the non-linearity is removed. Supplementary Fig. 1b shows similar plots from all tools except MaxQuant, which does not calibrate fragment masses. mzRefiner and MetaMorpheus correct the deviation to some extent, but MSFragger results in the smallest and most consistent mass error.

After mass calibration, fragment mass tolerance and the number of most intense peaks used (top-N) can both be updated to reduce the chance of matching noisy peaks. Taking advantage of MSFragger’s fast search speed, we developed a parameter optimization procedure (see “Methods” for details) to try different values for these settings and find the best-performing ones for the analysis at hand.

We used three Orbitrap datasets and one Bruker timsTOF PASEF dataset to test parameter optimization performance. The files used in this section can be found in Supplementary Data 1, and MSFragger parameters used in the database searches are described in “Methods”. To test the automatic parameter optimization procedure, we performed separate searches (after mass calibration) with each combination of fragment match tolerance and number of top-N peaks mentioned in “Methods”. Then, we compared the results to a search with parameter optimization turned on. Combinations of six fragment mass tolerances and five values of top-N peaks were tested. Figure 2c shows the number of PSMs from each of the four datasets with spectrum FDR < 0.01. A benchmark search without calibration or parameter optimization was also performed with 20 ppm fragment mass tolerance and 100 top-N peaks. The numbers of PSMs from the benchmark search for each dataset were 666,052 (PXD001468), 176,290 (PXD004940), 175,537 (PXD006675), and 459,608 (PXD010012).

Mass calibration alone improves search results for every dataset, which can be seen by comparing the benchmark values to the cells corresponding to 20 ppm fragment mass tolerance and 100 top-N peaks in Fig. 2c. The black boxes show that the automatic parameter optimization procedure successfully finds settings leading to high sensitivity in all cases. On average, the relative gain in PSMs (i.e., the difference divided by the benchmark value) from mass calibration coupled with parameter optimization is 10%.

### Sensitivity and precision of localization-aware open search

We tested the ability of the localization-aware open search to detect peptides with both simulated and real modifications. A dataset containing 24 fractions from Chick et al.^5^ (PXD001468) was used to test the ability of each open search tool to correctly identify random amino acid substitutions by generating a simulated dataset according to the procedure detailed in “Methods”. Briefly, roughly half of the spectra in this dataset were unmodified while the other half contained a single random amino acid replacement, representing unknown modifications. A high confidence list was first curated by taking PSMs found by both Comet^26^ and MS-GF+^27^. Then, we randomly picked half of the high confidence list for single amino acid replacement. Using the high confidence list as ground truth, we classified the results of the four tools, i.e., MSFragger (version 2.2, with regular OS and LOS), MetaMorpheus (version 0.0.303, with and without deconvolute precursors), pFind3^8^ (version 3.1.5, with and without mixture spectra), and TagGraph^9^ (version 1.8), into the three types: Type 1 (the same or similar sequence was identified), Type 2 (a different peptide was identified but it was found in another scan from the high confidence list), and Type 3 (a different peptide was identified and that sequence was not found in the list of high confidence PSMs). Leucine and isoleucine were considered the same for this purpose since they have identical mass. We observed that some spectra resulted in the same score for a peptide and its substring (Supplementary Data 2), and such cases were classified as Type 1.

**Fig. 3:**
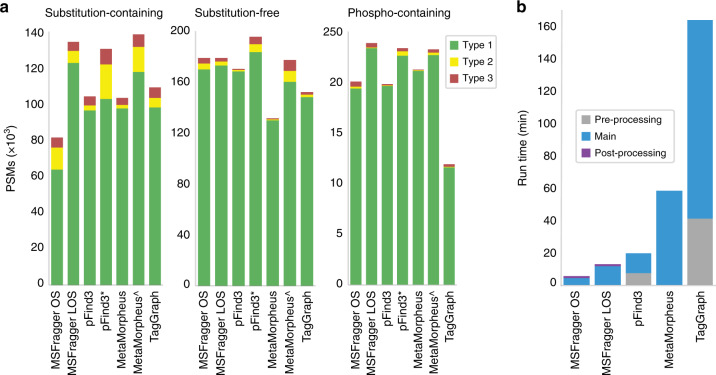
Comparison of search results and run times across tools. **a** Numbers of identified PSMs from MSFragger open search (MSFragger OS), MSFragger with localization-aware open search (MSFragger LOS), pFind3, pFind3 with mixture spectra (pFind*), MetaMorpheus, MetaMorpheus with precursor deconvolution (MetaMorpheus^), and TagGraph. Green indicates PSMs that agree with the high confidence list (Type 1). Yellow indicates PSMs with a different sequence, but one identified by other spectra in the high confidence list (Type 2). Red indicates spectra with a sequence that is not found in the high confidence list (Type 3). Left: numbers of substitution-containing PSMs from searching the simulated data. Middle: numbers of substitution-free PSMs from searching the simulated data. Right: numbers of phospho-containing PSMs from the phosphorylation-enriched data. **b** Average time (in minutes) taken by each tool to perform open search analysis on one representative mzML file.

There were 211,415 substitution-containing and 197,377 substitution-free PSMs in the high confidence list. The numbers of substitution-containing and substitution-free PSMs identified by each tool are shown in Fig. 3a. PSMs were filtered with spectrum FDR < 0.01. With shifted ion indexing and matching, MSFragger LOS identified about 65% more substitution-containing PSMs (134,413 vs. 81,648), with only a minimal loss of substitution-free PSMs (178,958 vs. 178,966) compared with MSFragger with regular OS. The precision (defined here as the fraction of the total reported PSMs that are Type 1 or Type 2, i.e., likely correct) of substitution-containing and substitution-free PSMs also increased, from 0.9294 to 0.9623 and 0.9755 to 0.9838, respectively. Note that most of the improvement can be attributed to the use of shifted ions and localization-aware open search, with smaller additional gain resulting from the mass calibration and parameter optimization steps (Supplementary Fig. 2). The mixture spectra feature of pFind3 and the deconvolute precursors feature in MetaMorpheus duplicate a spectrum into multiple spectra with different precursor masses. Without considering those additional spectra, MSFragger identified the most PSMs among all tools. It is also important to note that both pFind3 and MetaMorpheus restrict the delta masses to a predefined list (e.g., UniMod^14^), which is different from MSFragger’s unrestricted open search approach. pFind3 with mixture spectra and MetaMorpheus with deconvolute precursors produce more Type 2 PSMs than other tools. This likely because most spectra belonging to the Type 2 category are chimeric, as supported by analysis of precursor ion purities (Supplementary Fig. 3). The peptide index file from this search is roughly 72 MB.

A phosphorylation-enriched dataset containing six LC-MS files from Espadas et al.^28^ (PXD004940) was used to assess recovery of phosphorylated peptide identifications. From this dataset, we selected high confidence phosphorylated PSMs by taking the PSMs identified by both Comet and MS-GF+ (“Methods”), resulting in a list of 26,878 high confidence PSMs from 5796 phosphorylated peptides. We then used MSFragger (version 2.2, with regular OS and LOS) to analyze the dataset and compared with MetaMorpheus (version 0.0.3030, with and without deconvolute precursors), pFind3 (version 3.1.5, with and without mixture spectra), and TagGraph (version 1.8). The searches were performed without specifying phosphorylation or oxidation as variable modifications. PSMs were filtered with spectrum FDR < 0.01. To investigate the ability of each tool to correctly identify phosphorylated peptides, we compared the sequence identified for each spectrum to the high confidence list, again classifying each into the three types mentioned previously. Figure 3a shows that MSFragger LOS identifies ~19% more PSMs than with regular OS (23,828 vs. 20,072), resulting in the largest number of PSMs among all tools. The precision also increased slightly from MSFragger regular OS to LOS (0.9838 vs. 0.9728). Supplementary Fig. 2 shows that mass calibration and parameter optimization also contribute to improve performance of MSFragger LOS. The peptide index file from this search is roughly 1 GB.

### Application to large-scale HEK293 cell lysate data

**Fig. 4:**
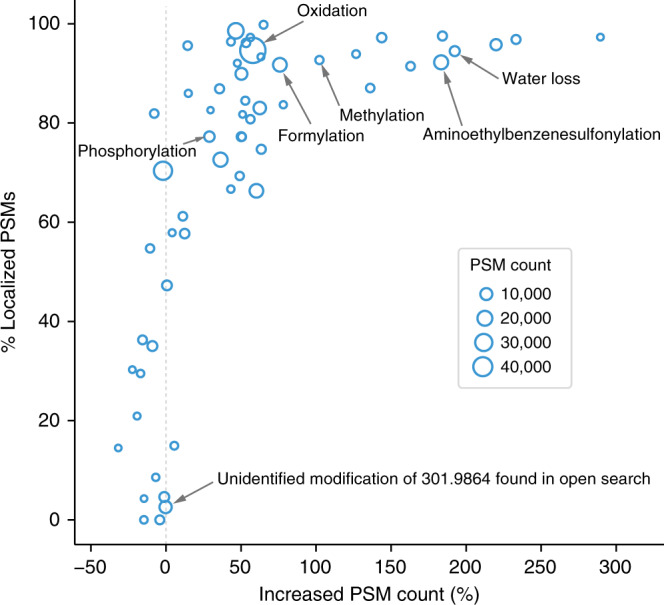
Percentage of localized PSMs vs. relative PSM increase in MSFragger LOS vs MSFragger regular OS search. Circle size indicates the number of PSMs with the corresponding delta mass.

We used a large-scale dataset^5^ from HEK293 cell lysate to demonstrate that localization-aware open search can find more PTMs, analyzing the dataset with MSFragger (version 2.2) with regular OS and LOS. PTM-Shepherd^29^ (version 0.2.14) was used to summarize the delta masses identified from the FDR-filtered search results from both MSFragger regular OS and MSFragger LOS. Given a delta mass and all corresponding PSMs, PTM-Shepherd reports the number of PSMs where the mass shift could be successfully localized. It also reports the number of PSMs from MSFragger regular OS and LOS searches. These reports are found in Supplementary Data 3. From the summarized delta masses, we made a scatter plot (Fig. 4) depicting the relationship between localization rate and percentage of changed PSM counts from MSFragger regular OS to MSFragger LOS. Delta masses outside of [−3.5, 3.5] Da with at least 200 PSMs were included.

Overall, MSFragger LOS finds more PSMs from almost all delta masses. PSMs with oxidation, methylation, formylation, water loss, and aminoethylbenzenesulfonylation increased by over 50%, while the increase in PSMs with phosphorylation was smaller due to its labile nature (and thus few fragments containing the modification). Spectra corresponding to PSMs with the uncharacterized delta mass of 301.986 Da had virtually no shifted fragments^15^, resulting in a small loss in the number of PSMs for this modification in MSFragger LOS. Similarly, we observed a slight decrease in the identification sensitivity of MSFragger LOS toward modification-free PSMs (Supplementary Data 3). This was expected, since such PSMs do not benefit from the inclusion of shifted ions in scoring. We also observed that MSFragger LOS helped to identify the correct delta mass/sequence combinations, alleviating some of the previous criticism of the open search strategy^11^. For example, the MSFragger regular OS found 407 PSMs with a ~189.047 Da delta mass, most of them placed on the N-terminus (Supplementary Data 3). The correct interpretation of these PSMs is N-terminal acetylation in combination with methionine oxidation (Supplementary Fig. 4). With MSFragger LOS, the number of such incorrectly interpreted PSMs was reduced to just 26.

### MSFragger LOS maintains fast search speed

We compared the open search run times of MSFragger (version 2.2), MetaMorpheus (version 0.0.303), pFind3 (version 3.1.5), and TagGraph (version 1.8) using eight fractions from Doll et al.^23^ (PXD006675), which contain ~80,000 tandem mass spectra in each run. All tasks were run on a desktop workstation with Intel Core i7-8700 (6 CPU cores, 3.2 GHz) with 32 GB memory. All tools were set to use 12 logical cores, except for TagGraph, which only supports single threading. We did not use the deconvolute precursors and mixture spectra options in MetaMorpheus and pFind3 for fair comparison. Note that both pFind3 and MetaMorpheus limit the searched delta masses to a predefined list (e.g., UniMod), while MSFragger searches the whole delta mass range.

Figure 3b shows the average time (in minutes) taken by each tool to analyze one mzML file. Also listed is the run time of MSFragger without mass calibration, parameter optimization, or shifted ion index. MSFragger uses Philosopher^30^ (2.0.0) to perform validation via PeptideProphet and generate reports, which we categorize as post-processing. pFind3 needs pParse^31^ to preprocess spectral files and TagGraph requires de novo sequencing result from PEAKS X^32^ as input, and we categorized these tasks as pre-processing. Though pFind3 was also able to analyze a single mzML file in 20 min, MSFragger had the shortest run time overall.

## Discussion

In summary, searching MS/MS spectra for modified peptides has traditionally been challenging due to large search space, with long computational analysis times and increased rate of false matches. In our earlier work we addressed this challenge with a highly efficient fragment ion indexing strategy and MSFragger software. Here we further extended our strategy with the introduction of a shifted ion index and localization-aware open search, providing a significant boost in the number of identified modified peptides. We have also implemented additional improvements to MSFragger software, such as mass calibration and fast parameter optimization. Although here we focused on modified peptides only, we found that other applications of MSFragger, including conventional closed searches and mass offset searches for specific PTMs such as N-linked and O-linked glycosylation, benefit significantly from these developments as well.

## Methods

### Datasets

The datasets used here were downloaded from ProteomeXchange^33^, with the accession numbers PXD001468 (HEK293 cell lysate; Q-Exactive Orbitrap mass spectrometer)^5^, PXD004940 (HeLa cell lysate; Orbitrap Fusion Lumos mass spectrometer)^28^, PXD006675 (human heart tissue samples; Q-Exactive HF Orbitrap mass spectrometer)^23^, and PXD010012 (HeLa cell lysate; timsTOF Pro mass spectrometer)^34^. Supplementary Data 1 describes the selected files used from each dataset. Details regarding sample preparation and data acquisition for each dataset can be found in the corresponding publications. Files in mzML spectral format were used for all experiments except for the mass calibration comparison, where the vendor’s raw format was used as it works best for MaxQuant.

### Precursor and fragment mass calibration

We developed a supervised nonparametric approach to calibrate precursor and fragment masses. MSFragger first searches the spectral file according to the parameters (with slight changes) and database provided by the user. It takes high scoring PSMs (those with expectation value below 0.001) and divides them into two equal-sized sets: the first set is used to build both precursor and fragment mass profiles and the second (validation) set is used to evaluate the performance of the calibration. To build a precursor profile, MSFragger traces MS1 peaks to get three extracted ion chromatograms (XICs) corresponding to 0, +1, and +2 ^13^C isotopes for each precursor ion. To trace each isotope’s XIC, MSFragger first collects all relevant peaks around the precursor *m/z* and retention time. Then, it performs a Gaussian smoothing and tries to link adjacent peaks starting from the PSM’s precursor peak. It also tries to find boundaries using a logic similar to MaxQuant^24^: the intensity of a boundary needs to be smaller than *c* × min(*I*_1_,*I*_2_), where *c* is a constant (0.5 in MSFragger), *I*_1_ and *I*_2_ are the intensities of the adjacent apexes. After tracing the XICs, MSFragger calculates an intensity-weighted mass $d ^{\prime} _{t,{m_{t}}}$ corresponding to retention time *t* and observed *m/z* value *m*_*t*_, using the following equation:

1 $$ {d^{\prime}_{t,mt} = \frac{1}{3}\mathop {\sum }\limits_{i = 0}^2 \frac{{\mathop {\sum }\nolimits_{j = l}^u \left( {m_{i,j} - m_t - i \times 1.0033548378} \right) \times I_{i,j}}}{{\mathop {\sum }\nolimits_{j = l}^u I_{i,j}}}} ,$$

where *i* is the number of the ^13^C isotopic peak, *l* is the first-traced peak index, *μ* is the last-traced peak index, *m*_*i,j*_ is the *m/z* value of *j*th peak from *i*th XIC, *m*_*t*_ is the precursor *m/z* at retention time *t*, and *I*_*i,j*_ is the intensity of *j*th peak from *i*th XIC.

Then, the total relative deviation can be calculated as

2 $$ {d_{t,m_t} = \frac{{10^6 \times \left( {m_t - m_{\mathrm{c}} + d^{\prime}_{t,m_t}} \right)}}{{m_{\mathrm{c}}}}} ,$$

where $d ^{\prime} _{t,{m_{t}}}$ is the total relative deviation (in ppm) at retention time *t* and precursor *m*_*t*_; *m*_*c*_ is the theoretical *m/z* according to the matched peptide sequence.

Intuitively, $d ^{\prime} _{t,{m_{t}}}$ can be treated as the deviation from an ion’s measured *m/z* to the precursor *m/z* stored in one of the ion’s MS/MS scans. The numerator of the right side of Eq. (2) can be treated as the deviation from an ion’s measured *m/z* to its theoretical *m/z*. Thus, $d _{t,{m_{t}}}$ is the systematic relative deviation at retention time *t* and *m/zm*_*t*_.

With $d _{t,{m_{t}}}$, MSFragger builds a profile, which is in the form of a *X*-by-*Y* matrix ***P*** reflecting the relative deviation over the whole range of retention time and *m/z*. In order to avoid overfitting, the retention time and *m/z* are divided into coarse grids, which results in *X* = ceil(*max*(*t*)/5) and *Y* = ceil(*max*(*m*_*t*_)/200). The index of the cell is [floor(*t*/5),floor(*m*_*t*_/200)]. Each $d _{t,{m_{t}}}$ contributes to its neighboring cells with weights as illustrated in Fig. 1c. The upper plane is a duplicate of the blue cell in the lower plane, and the other three orange cells represent its neighbors. The black dot in the top plane is the location of $d _{t,{m_{t}}}$ (*d* in the figure), which is assigned to the four cells (lower plane in Fig. 1c) weighted by the normalized distances to the boundaries of the neighboring cells (*x* and *y*). Similarly, another *X*-by-*Y* matrix is created to record the total weight of each cell received. The final mass deviation of a cell is the weighted mass deviations from the first matrix divided by the total weight from the second matrix. Take a scan with retention time located at 2nd row and precursor *m/z* located at 5th column for example. Its precursor mass error is 10 ppm, normalized distance to the horizontal boundary is 0.3, and normalized distance to the vertical boundary is 0.5. For the first matrix, 10 × (1 − 0.3) × (1 − 0.5) ppm is added to the cell with coordinate [2, 5], 10 × (1 − 0.3) × 0.5 ppm is added to the cell with coordinate [2, 6], 10 × 0.3 × (1 − 0.5) ppm is added to the cell with coordinate [3, 5], and 10 × 0.3 × 0.5 ppm is added to the cell with coordinate [3, 6]. Similarly, (1 − 0.3) × (1 − 0.5), (1 − 0.3) × 0.5, 0.3 × 1 − 0.5, and 0.3 × 0.5 are added to [2, 5], [2, 6], [3, 5], and [3, 6] cells in the second matrix. After processing all selected scans, MSFragger divides each cell in the first matrix by the corresponding cell in the second matrix to get the final mass deviation of the profile.

Since each element in the profile reflects the relative deviation from an ion’s measured *m/z* to its theoretical *m/z*, given *m*_*t*_ from one of the ion’s tandem mass spectra, the calibrated $\hat m_t$ can be calculated using

3 $${\hat m_t = \frac{{m_t}}{{1 + \left( {P_{ \lfloor t/5 \rfloor,\lfloor m_t/200 \rfloor} - \frac{{d{\prime}_{t,m_t} \times 10^6}}{{m_{\mathrm{c}}}}} \right) \times 10^{ - 6}}}} .$$

In Eq. (3), $\left( {P_{\lfloor t/5 \rfloor, \lfloor m_t/200 \rfloor} - \frac{{d{\prime}_{t,m_t} \times 10^6}}{{m_{\mathrm{c}}}}} \right)$ is equivalent to the relative deviation from the precursor *m/z* to the theoretical *m/z*.

If there were no MS1 scans in the given spectral data (e.g., MGF file were used as input), MSFragger would skip the peak tracing step and Eq. (1). Thus, Eq. (2) could be simplified to

4 $$ {d_{t,m_t} = \frac{{10^6 \times \left( {m_t - m_{\mathrm{c}}} \right)}}{{m_{\mathrm{c}}}}},$$

and Eq. (3) could also be simplified to

5 $${\hat m_t = \frac{{m_t}}{{1 + P_{\left\lfloor {t/5} \right\rfloor ,\left\lfloor {m_t/200} \right\rfloor } \times 10^{ - 6}}}} .$$

Building a fragment profile and calibrating the fragment *m/z* are similar to the above approaches except that there is no peak tracking step, and Eqs. (4) and (5) are used in profile building and mass calibration.

After calibrating precursor *m/z* and fragment *m/z* for all scans, MSFragger uses the validation set to estimate the median and median absolute deviation (MAD) of mass deviation. Since the calibration profile is built solely using the first set, the estimated median and MAD can be treated as an independent evaluation of the mass calibration accuracy.

### Parameter optimization procedure

After calibrating the precursor *m/z* and fragment *m/z*, the fragment mass tolerance can be narrowed to reduce random matches without sacrificing sensitivity. The number of topmost intense peaks retained for matching can also be adjusted accordingly. We noticed that the standard deviation (SD) and MAD of the mass errors were sensitive to the initial search tolerances. Wider initial tolerances, which contain more outliers, resulted in larger SD and MAD. Taking advantage of MSFragger’s fast searching speed, we developed an empirical approach to find better parameters through simplified searches on the spectra (i.e., first search).

We preset a certain number of candidate fragment mass tolerances for high and low mass resolution MS/MS data. In the version of MSFragger used in this work, these values were set to 7, 10, 15, 20, 25, and 30 ppm; and 100, 200, 300, 400, and 500 ppm for high and low mass accuracy MS/MS spectra, respectively. We also predefine possible numbers of peaks to be used for matching, values of 100, 125, 150, 175, and 200 here. Instead of trying all possible combinations of these parameters (e.g., 6 × 5 or 5 × 5), we assumed that the effects of the parameters were independent of each other, an assumption we verified with numerous independent experiments. With this assumption, MSFragger tries different parameters sequentially and picks the combination with the highest estimated sensitivity (number of identifications at 1% spectrum FDR, estimated internally by MSFragger using the expectation value as the only score and the target-decoy strategy). We also found that there is only one maximum in the sensitivity with respect to a particular parameter. Thus, for each parameter, MSFragger skips the rest of the candidate parameters after a maximum estimated sensitivity is achieved. MSFragger also checks whether performing intensity transformation (square root) and/or removing precursor peaks prior to computing the hyperscore improves the annotation result. This process requires multiple repeated searches (up to 13 searches using the tested parameter settings described). However, this can be done quickly for most searches, or can be turned off for time-consuming searches such as nonspecific digestion. Finally, MSFragger uses the updated parameters to perform the main search.

### Shifted ion indexing, matching, and delta mass localization

Both regular and shifted fragment ion indexes are generated in advance given a sequence database. The shifted ion index is generated as follows: suppose there is an unknown-modification-containing MS/MS spectrum with precursor mass *p*_*t*_ and shifted peaks with masses *p*_*i*_, where *i* ϵ [1,*M*]. Given a peptide with total mass *m*_*t*_ and regular (not containing the unknown modification) fragment ion masses *m*_*j*_ where *j* ϵ [1,*N*], we can assume

6 $$\begin{array}{*{20}{c}} {p_t - m_t \approx p_i - m_j} \end{array},$$

where ≈ indicates that a tolerance (e.g., 20 ppm) is applied to this equivalence. The regular fragment ion can be either N-terminal ions or C-terminal ions. Equation (6) can be easily transformed to

7 $$\begin{array}{*{20}{c}} {p_t - p_i \approx m_t - m_j} \end{array}.$$

Equation (7) shows that we can match a shifted peak in the experimental spectrum against a regular theoretical ion by subtracting the peak’s mass from its precursor mass as long as we perform a similar transformation (Fig. 1a). With this strategy, we can decouple the spectrum-wise process from the database-wise process, which enables us to build a shifted ion index before processing any spectral data. Given an MS/MS spectrum, MSFragger matches experimental peaks against a regular ion index, then against a shifted ion index. With regular ion matching coupled with shifted ion matching, the final outcome is the same as matching the experimental spectrum against a theoretical spectrum with certain peaks shifted according to the mass difference between the precursor mass and the calculated peptide mass. Because the indexes are built in advance, the matching and scoring can be performed rapidly.

The shifted ion index adds peaks to each spectrum comparison. Although most of the added peaks are not noise, the chance of random matches may still be increased due to increased search space. We proposed a workflow to coordinate shifted ion matching with regular ion matching (Fig. 1b) to mitigate this potential increase in false fragment ion matches. MSFragger records the matched peaks from regular and shifted ion matching during the search. Then, it calculates hyperscores using the peaks from the regular ion matching only and picks the top-scoring candidate. If the mass difference between the precursor mass and the candidate’s calculated mass falls outside a predefined range (−1.5 to 3.5 Da by default), MSFragger calculates another list of hyperscores by combining the peaks from both regular and shifted ion matching. In this way, MSFragger tries all possible modified locations one-by-one by taking the regular ion matched peaks up to that location and shifted ion matched peaks from that location. In the meantime, MSFragger also tries to avoid double counting caused by overlapped matches. Since MSFragger has already recorded all matched peaks beforehand, this procedure can be processed very fast. The higher scoring one from the two top candidates (one from regular ion matching only, the other from both regular and shifted ion matching) are selected as the final hit. As part of the process, MSFragger also localizes the delta mass to the most probable location(s) within the identified peptide. It takes the location(s) with the highest score as the localized site(s). MSFragger reports the localization results, including a delta score between the best and the second-best localized residues, in a tab-delimited (tsv) file. This delta score can be used as a confidence measure of localization.

### Mass calibration and parameter optimization analysis

We used eight fractions from Doll et al.^23^ (PXD006675) to demonstrate the performance of mass calibration. MaxQuant^24^ (1.6.10.43), mzRefiner^25^ (from ProteoWizard 3.0.19311 64-bit), and MetaMorpheus^35^ (0.0.303) were used for comparison. We observed that MaxQuant and MetaMorpheus performed better with the raw file format, so we used raw files for all tools in this comparison. MaxQuant reports the calibrated precursor mass error only for FDR-filtered PSMs in the msms.txt file. Thus, for a fair comparison, we compared only this set of scans for all tools. MaxQuant calibrates precursor masses only. For the other three tools, both precursor mass and fragment mass calibration were evaluated. Since all eight fractions gave similar results, we only show the result from fraction “20160901_QEp2_SoDo_SA_LC12-13_PV8-frac3” for simplicity.

We used the datasets listed in Supplementary Data 1 to demonstrate the performance of MSFragger’s parameter optimization procedure. All analyses in this manuscript were performed using MSFragger (version 2.2). We first used MSFragger to analyze these datasets without mass calibration and parameter optimization as a benchmark. A database of reviewed human proteins and common contaminants from UniProt^36^ (downloaded on Sep. 30, 2019; 20463 proteins) was used. Decoy proteins were generated and added to the database by reversing the target proteins. The benchmark run fragment mass tolerance was set to 20 ppm, and number of top-N peaks used for matching was set to 100. The precursor tolerance range was set to (−150 to 500 Da). ^12^C/^13^C isotope errors were not allowed (set to 0), which is default for all open searches. The peptide mass range was set from 500 to 5000 Da, and peptides of length 7 to 50 residues were allowed. One missed cleavage per peptide was allowed. Acetylation of protein N-termini and oxidation of methionine were specified as variable modifications. Carbamidomethylation of cysteine was specified as a fixed modification. The searches were performed in the MSFragger LOS mode (localize_delta_mass parameter set to 1). MSFragger search results were processed using PeptideProphet^37^ using the Philosopher toolkit (version 2.0.0), and PSM were filtered to 1% FDR using the target-decoy strategy implemented as part of the Philosopher filter command.

Comparing with the benchmark run (calibrate_mass parameter set to 0), MSFragger was also run with both mass calibration and parameter optimization turned on (calibrate_mass parameter set to 2), trying all combinations of different fragment mass tolerances and top-N peaks as described above. For each dataset, a 6 × 5 matrix was generated whose rows and columns correspond to different fragment mass tolerances and numbers of top-N peaks kept for matching, respectively (Fig. 2c).

### Analysis using simulated data

We generated a simulated dataset with peptides containing random amino acid substitutions (MS/MS spectra themselves were not modified in any way). This approach was used by Na et al.^10^ and Yu et al.^7^. We extensively improved it by making it closer to a real application and the confident list more reliable. We first generated a high confidence PSM list using Comet^26^ (version 2019012) and MS-GF+^27^ (version 20190703) using all 24 fractions from the HeLa data published by Chick et al.^5^ (PXD001468). In these searches, precursor mass tolerance was set to 50 ppm. A database of reviewed human proteins and common contaminants from UniProt^36^ (downloaded on September 30, 2019; 20463 proteins) was used. Decoy sequences were generated by reversing the target sequences. For Comet, the fragment bin tolerance was set to 0.02 Th. For MS-GF+, the instrument type was set to 3 (i.e., Q-Exactive). Acetylation of protein N-termini and oxidation of methionine were specified as a variable modification. Carbamidomethylation of cysteine was specified as a fixed modification. There was no isotope error correction applied. The peptide mass range was set from 500 to 5000 Da, and peptides with length 7 to 50 residues were allowed. One missed cleavage per peptide was allowed. Both Comet and MS-GF+ search results were processed using PeptideProphet^37^ and ProteinProphet^38^ using the Philosopher^30^ toolkit. PSMs were filtered using both spectrum and protein FDR of 1% using Philosopher filter command to generate a high confidence PSM list. This nonredundant peptide list was split into two subsets. In the first, one amino acid in each peptide sequence was substituted with a different residue to simulate a modification-containing peptide (substitution-containing subset). The second subset remained unchanged (substitution-free subset). All corresponding sequences were modified in the original protein sequence database accordingly. There were 211,415 substitution-containing and 197,377 substitution-free PSMs in the high confidence list at 1% PSM and protein FDR.

We used MSFragger (version 2.2), MetaMorpheus (version 0.0.303), pFind3 (version 3.1.5), and TagGraph (version 1.8) to search the same 24 spectral files against the modified database. MSFragger’s precursor mass lower and upper bounds were set to −150 and 500 Da, respectively. We used calibrate, GPTMD, and search tasks in MetaMorpheus. The maxdelta parameter in pFind3 was set to 500 by default. Protein N-terminal acetylation was set as a variable modification, and carbamidomethylation of cysteine was specified as a fixed modification. In all tools, the precursor and fragment mass tolerances were set to 20 ppm. The peptide mass range was set from 500 to 5000 Da, and peptides with length between 7 and 50 residues were allowed. One missed cleavage per peptide was allowed. For MSFragger, this resulted in a fragment ion index of roughly 72 MB. MSFragger was run with regular OS and LOS, governed by the localize_delta_mass parameter set to 0 or 1, respectively. For MetaMorpheus, pFind3, and TagGraph, the search results were filtered to 1% spectrum FDR using the FDR estimation and filtering modules of those tools. For MSFragger, the results were processed using Philosopher as described in the last section. We observed that when the mixture spectra option was enabled in pFind3, multiple peptides could be assigned to a potentially chimeric spectrum, which resulted in more than one PSM for a single spectrum. MetaMorpheus also has a similar option called deconvolute precursors. Thus, pFind3 and MetaMorpheus searches were also repeated with these options enabled, allowing multiple peptide annotations per spectrum.

The FDR-filtered PSM lists from each tool (from the modified database search) were compared against the high confidence list generated using Comet and MS-GF+. PSMs were classified into three types: Type 1 (same peptide identification allowing exact substrings since such cases resulted in the same scores in most cases, see Supplementary Data 2), Type 2 (a different sequence was identified but it was found in another PSM in the high confidence list), and Type 3 (a different sequence was identified that was not found elsewhere in the high confidence list). In the sequence comparison, leucine and isoleucine were treated the same since they have the identical mass. We assumed that PSMs of Type 1 or Type 2 are likely correct, and calculated, for each tool, the precision as the fraction of all PSMs in the list that were either Type 1 or Type 2.

### Analysis using phosphopeptide-enriched data

We used six files from a phosphopeptide-enriched experiment by Espadas et al.^28^ (PXD004940). We first obtained a high confidence PSM list using conventional Comet (2019012) and MS-GF+ (20190703) searches. Precursor mass tolerance was set to 50 ppm. A database of reviewed human proteins and common contaminants from UniProt^36^ (downloaded on Sep. 30, 2019; 20463 proteins) was used. Decoy sequences were generated by reversing the target sequences. For Comet, the fragment bin tolerance was set to 0.02 Th. For MS-GF+, the instrument type was set to 3 (i.e., Q-Exactive). Acetylation of protein N-termini, oxidation of methionine, and phosphorylation of serine, threonine, and tyrosine were specified as variable modifications. Remaining parameters and FDR filtering steps were identical to those in the simulated dataset analysis. These search parameters resulted in an MSFragger peptide index file of ~1 GB on disk. Phosphorylated PSMs identified by both Comet and MS-GF+ were used to generate a high confidence list of phosphopeptides. We selected a list of 26,878 high confidence PSMs from 5796 phosphorylated peptide sequences. We then used MSFragger (version 2.2, regular OS and LOS mode), MetaMorpheus (version 0.0.303), pFind3 (version 3.1.5), and TagGraph (version 1.8) to perform OSs on the same six spectral files, without setting phosphorylation or oxidation as a variable modification. For each tool, the PSMs passing the FDR filters were classified into one of the three types, and the precision was calculated as described above.

### Large-scale PTM analysis using fractionated HEK293 data

We performed two open search analyses using HEK293 cell lysate data published by Chick et al.^5^ (PXD001468), one with MSFragger regular OS and the other with MSFragger LOS. The precursor mass lower and upper bounds were set to −150 and 500 Da, respectively. Acetylation of protein N-terminus was specified as a variable modification. Carbamidomethylation of cysteine was specified as a fixed modification. A database of reviewed human proteins and common contaminants from UniProt^36^ (downloaded on September 30, 2019; 20463 proteins) was used. Decoy proteins were generated by reversing the target proteins. The precursor and fragment mass tolerance were set to 20 ppm. The peptide mass range was set from 500 to 5000 Da, and peptides with between 7 and 50 residues were allowed. One missed cleavage per peptide was allowed. Philosopher^30^ (version 2.0.0) coupled with PeptideProphet^37^ and ProteinProphet^38^ were used to estimate FDR. PSMs were filtered with spectrum FDR <0.01 and protein FDR <0.01. We then used PTM-Shepherd^29^ to summarize the delta masses observed from the filtered PSMs. Delta masses were annotated if they fell outside the [−3.5, 3.5] Da range and had at least 200 PSMs.

### Speed comparisons

Speed comparisons were performed using MSFragger (version 2.2, regular OS and LOS mode), MetaMorpheus (version 0.0.303), pFind3 (version 3.1.5), and TagGraph (version 1.8), each run on eight fractions from Doll et al.^23^ dataset containing ~80,000 MS/MS spectra in each fraction. All tasks were run on a desktop workstation with an Intel Core i7-8700 (12 logical cores, 3.2 GHz) CPU and 32 GB memory. All tools were set to use 12 logical cores, except for TagGraph, which only supports single threading. In this analysis, we did not use the deconvolute precursors and mixture spectra options in MetaMorpheus and pFind3 for fair comparison. For MSFragger, we performed regular OS and LOS analysis (for MSFragger LOS, with mass calibration and parameter optimization). For MSFragger analysis, post-search analysis using Philosopher (PeptideProphet, filtering, and report generation) was counted as post-processing time. pFind3 needs pParse^31^ to preprocess spectral files and TagGraph requires de novo sequencing result from PEAKS X^32^ as input. These tasks were categorized as pre-processing time.

### Reporting summary

Further information on research design is available in the Nature Research Reporting Summary linked to this article.

## Supplementary information

Supplementary Information

Description of Additional Supplementary Files

Supplementary Data 1

Supplementary Data 2

Supplementary Data 3

Supplementary Data 4

Reporting Summary

## Data Availability

Mass spectrometry data that support the findings of this study are available from ProteomeXchange (http://www.proteomexchange.org/) with the identifiers PXD001468, PXD004940, PXD006675, and PXD010012. Supplementary Data 1 describes the selected files used from each dataset.

## Code availability

The links to download MSFragger software, user guides, and tutorials are available at https://msfragger.nesvilab.org/. MSFragger can be run as a stand-alone tool, or via the FragPipe Graphical User Interface (https://fragpipe.nesvilab.org/).
