# Supplementary material for: Identification of modified peptides using localization-aware open search
Source: Nat Commun. 2020 Aug 13;11:4065. doi: 10.1038/s41467-020-17921-y (PMC7426425; doi:10.1038/s41467-020-17921-y)
Supplement: Supplementary file 1 — Supplementary Information [file 41467_2020_17921_MOESM1_ESM.pdf]

Identification of Modified Peptides using Localization-aware Open Search

Fengchao Yu[1], Guo Ci Teo[1], Andy T. Kong[1], Sarah E. Haynes[1], Dmitry M. Avtonomov[1], Daniel J. Geiszler[1], Alexey I. Nesvizhskii[1,2,*]

[1] Department of Pathology, University of Michigan, Ann Arbor, Michigan, USA

[2] Department of Computational Medicine and Bioinformatics, University of Michigan, Ann Arbor, Michigan, USA

*****Corresponding author: nesvi@med.umich.edu

Supplementary Figures:

Supplementary Figure 1: Mass calibration results from MaxQuant, mzRefiner, MetaMorpheus, and MSFragger.

Supplementary Figure 2: Numbers of identified PSMs from MSFragger with localization-aware open search but without mass calibration and parameter optimization (MSFragger LOS") and MSFragger with localization-aware open search (MSFragger LOS).

Supplementary Figure 3: Violin plots of ion purities of three PSM types from the simulation dataset.

Supplementary Figure 4: An example MS/MS spectrum annotated with two different peptides.

(a)

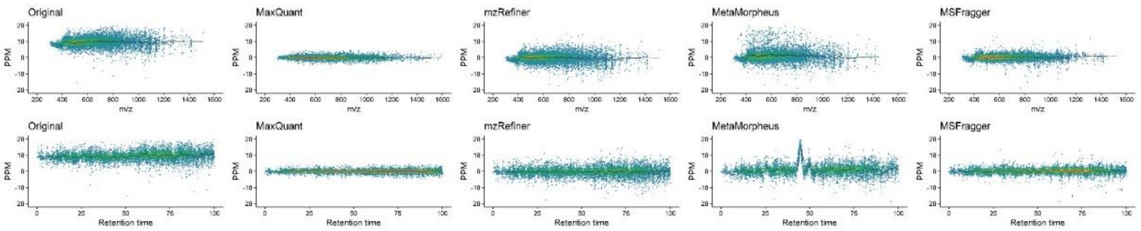

(b)

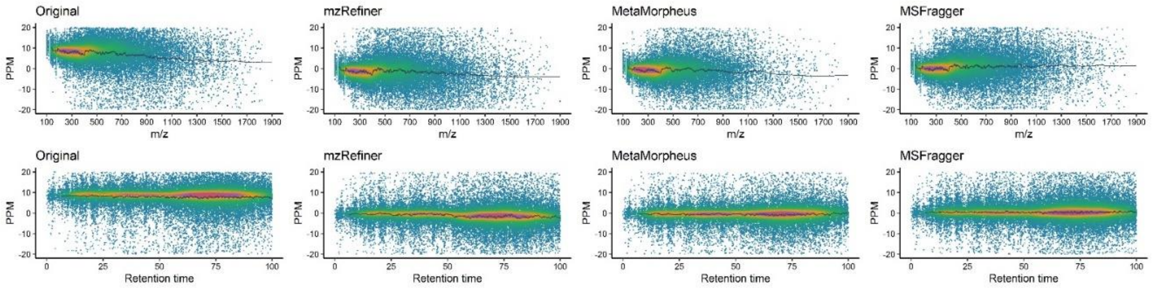

Supplementary Figure 1: Mass calibration results from MaxQuant, mzRefiner, MetaMorpheus, and MSFragger. (a) Precursor mass error before and after mass calibration. Precursor mass error is shown against m/z (upper panel) and retention time (lower panel), where color signifies point density. The smoothed moving average line is shown in dark grey. **(b)** Same as (a) for fragment mass calibration.

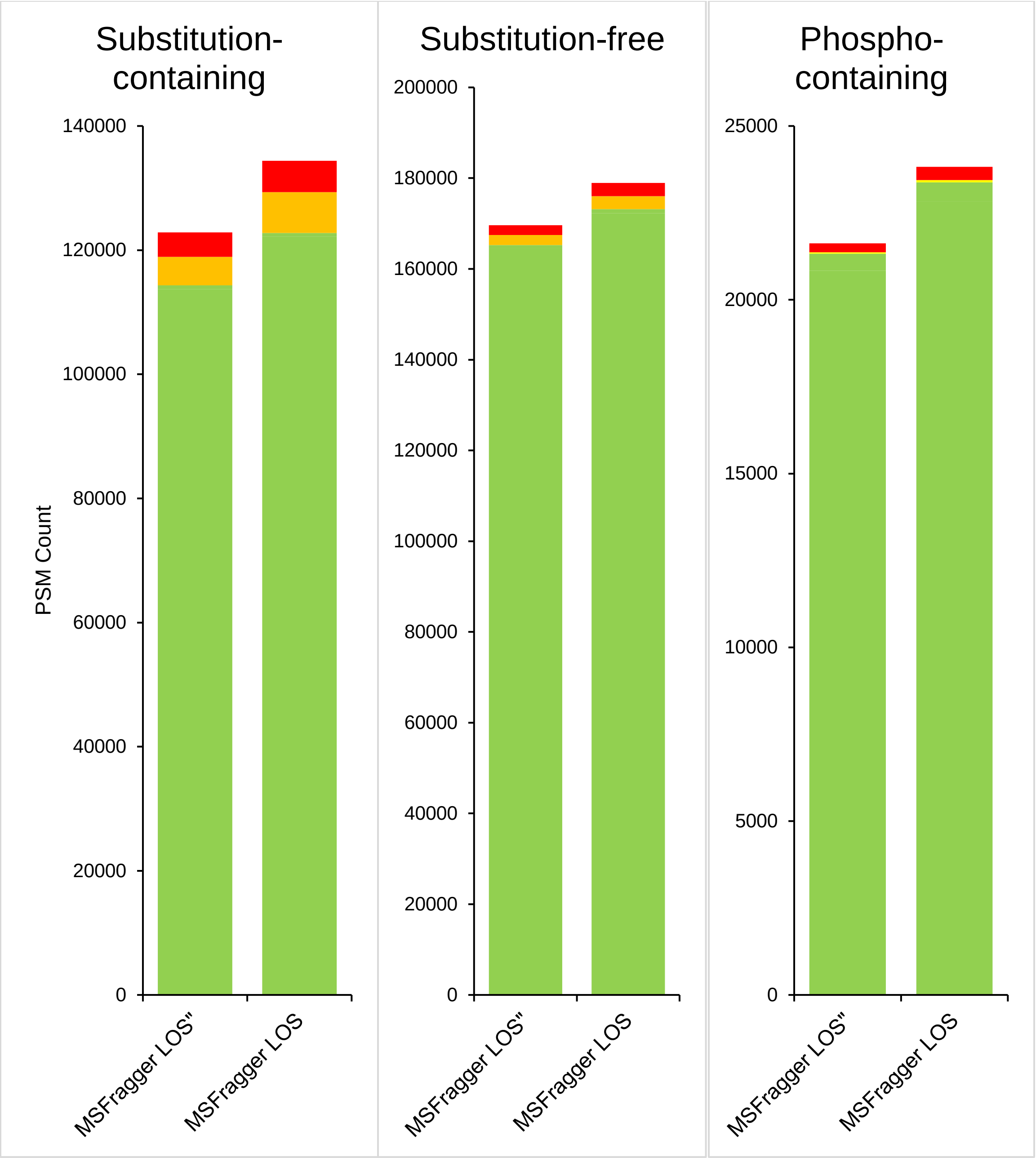

Supplementary Figure 2: Numbers of identified PSMs from MSFragger with localization-aware open search but without mass calibration and parameter optimization (MSFragger LOS") and MSFragger with localization-aware open search (MSFragger LOS). Green indicates PSMs that agree with the high confidence list (Type 1). Yellow indicates PSMs with a different sequence, but one identified by other spectra in the high confidence list (Type 2). Red indicates spectra with a sequence that is not found in the high confidence list (Type 3). Left: numbers of substitution-containing PSMs from searching the simulated data. Middle: numbers of substitution-free PSMs from searching the simulation data. Right: numbers of phospho-containing PSMs from the phosphorylation-enriched data.

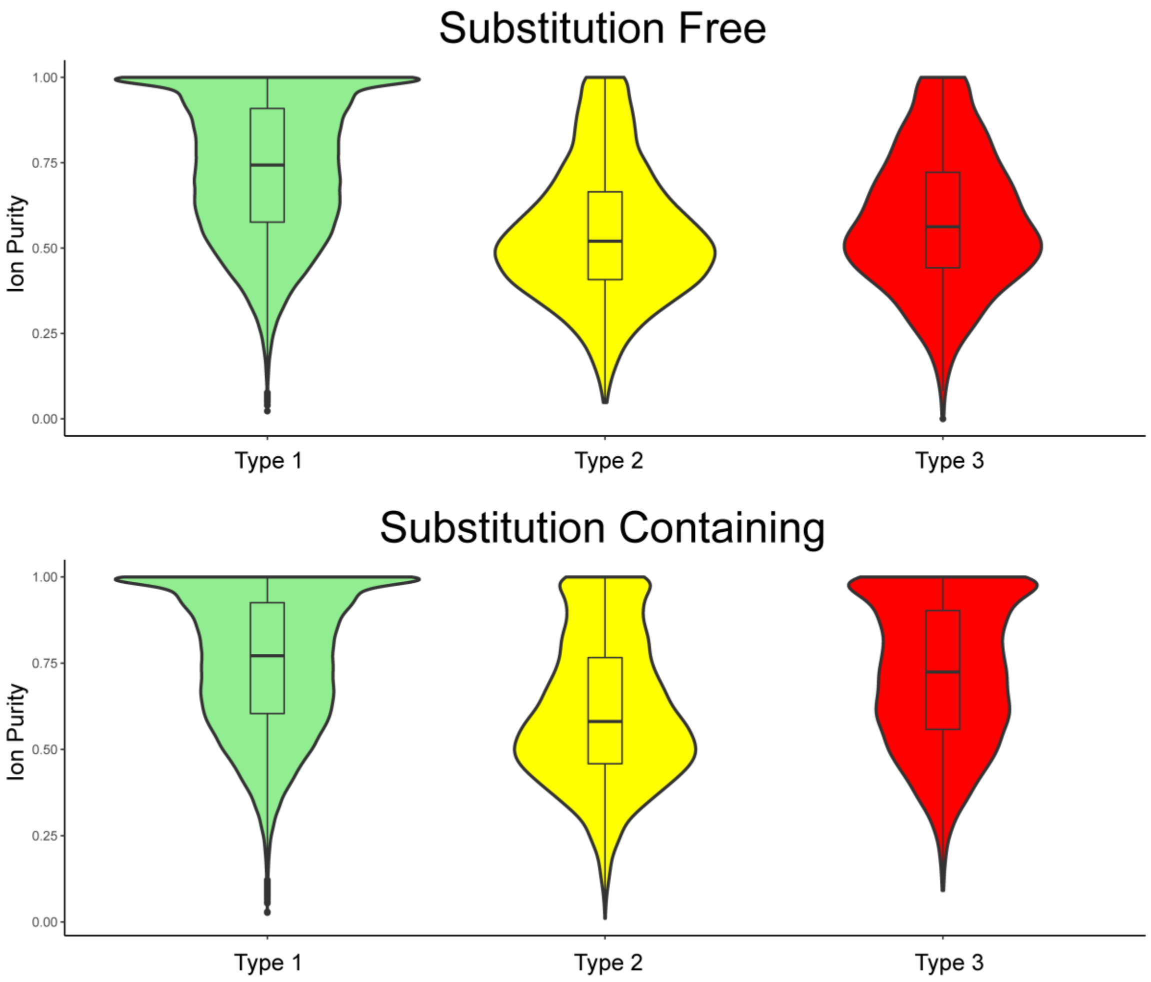

Supplementary Figure 3: Violin plots of ion purities of three PSM types from the simulation dataset. Ion purity is the intensity of the precursor peak divided by the summed intensity of all peaks in the isolation window. Green is from Type 1, yellow is from Type 2, and red is from Type 3. The distribution of Type 2 shows that most of the PSMs have a relatively low ion purity, which indicates that the spectra contain more than one peptide ions (i.e., chimeric spectra). Interior boxplots show the median (center line), lower and upper quartiles (box bounds), and the maximum and minimum ion purities (whisker bounds). n=173152 Type 1 substitution-free PSMs, n=2903 Type 2 substitution-free PSMs, n=663 Type 3 substitution-free PSMs, n=122767 Type 1 substitution-containing PSMs, n=6582 Type 2 substitution-containing PSMs, n=5064 Type 3 substitution-containing PSMs. Raw data corresponding to this figure can be found in **Supplementary Data 4**.

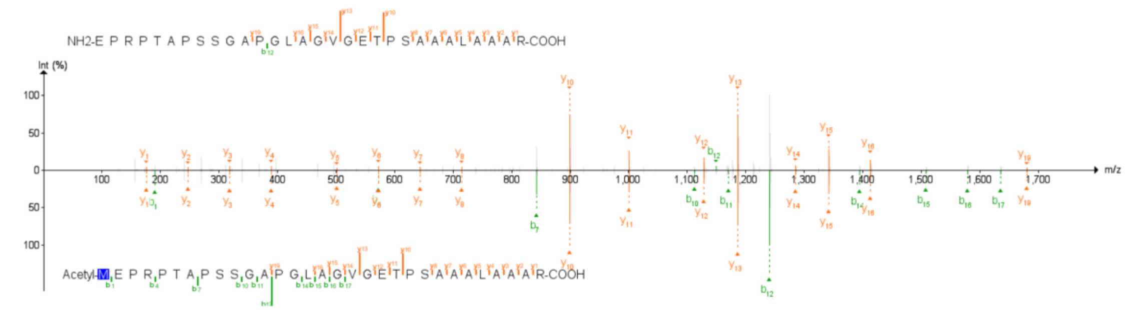

Supplementary Figure 4: An example MS/MS spectrum annotated with two different peptides. The top half of the spectrum was annotated by MSFragger OS and the lower half was annotated by MSFragger LOS.
