# Supplementary material for: Identification of modified peptides using localization-aware open search
Source: Nat Commun. 2020 Aug 13;11:4065. doi: 10.1038/s41467-020-17921-y (PMC7426425; doi:10.1038/s41467-020-17921-y)
Supplement: Supplementary file 2 — Description of Additional Supplementary Files [file 41467_2020_17921_MOESM2_ESM.pdf]

**Title:** Supplementary Data 1:
**Description:** List of all mass spectrometry runs (file names) used in this study.

**Title:** Supplementary Data 2:
**Description:** List of similar sequences (by substring) resulting in the same score. Six phosphorylation-enriched runs from Espadas et al. (2017) were used to generate this table.

**Title:** Supplementary Data 3:
**Description:** Output from PTM-Shepherd.

**Title:** Supplementary Data 4:
**Description:** Ion purity scores used in plotting Supplementary Figure 3. There are six tables corresponding to substitution-free Type 1, substitution-free Type 2, substitution-free Type 3, substitution-containing Type 1, substitution-containing Type 2, and substitution-containing Type 3 cases.
